# Pharmacophore feature-based virtual screening for finding potent GSK-3 inhibitors using molecular docking and dynamics simulations

**DOI:** 10.6026/97320630012391

**Published:** 2016-11-30

**Authors:** Navneet Chauhan, Anuradha Gajjar, Syed Hussain Basha

**Affiliations:** 1 Department of Pharmaceutical Chemistry, Institute of Pharmacy, Nirma University, Ahmedabad 382 481, Gujarat, India; 2 Department of Pharmaceutical Chemistry, Ramanbhai Patel College of Pharmacy, Charotar University of Science and Technology, Changa 388 421, Gujarat, India; 3 Innovative Informatica Technologies, Hyderabad 500 049, Telangana, India

**Keywords:** Docking, GSK-3, Molecular dynamics, Tideglusib, Virtual screening

## Abstract

Glycogen synthase kinase-3 (GSK-3) is a multitasking serine/threonine protein kinase, which is associated with the pathophysiology of several diseases such as diabetes, cancer, psychiatric and neurodegenerative diseases. Tideglusib is a potent, selective, and irreversible GSK-3 inhibitor that has been investigated in phase II clinical trials for the treatment of progressive supranuclear palsy and Alzheimer's disease. In the present study, we performed pharmacophore feature-based virtual screening for identifying potent targetspecific GSK-3 inhibitors. We found 64 compounds that show better GSK-3 binding potentials compared with those of Tideglusib. We further validated the obtained binding potentials by performing 20-ns molecular dynamics simulations for GSK-3 complexed with Tideglusib and with the best compound found via virtual screening in this study. Several interesting molecular-level interactions were identified, including a covalent interaction with Cys199 residue at the entrance of the GSK-3 active site. These findings are expected to play a crucial role in the binding of target-specific GSK-3 inhibitors.

## Background

Glycogen synthase kinase-3 (GSK-3) is a multitasking serine/threonine protein kinase. Structurally, GSK-3 is a twodomain kinase fold, with a β-strand domain and an α-helix domain. The β-strand domain (residues 25–138) is located at the N-terminal end and comprises seven antiparallel β-strands interrupted by a short helix (residues 96–102) comprising highly conserved residues. The Arg96 residue is involved in domain alignment, whereas the Glu97 residue that forms a salt bridge with Lys85 plays a crucial role in the catalytic activity of the enzyme. The α-helical domain comprises residues 139–343 forms at the C-terminal end. The catalytic site is located at the interface of the α-helical and β-strand domains and is surrounded by a hinge region and a glycine-rich loop. The activation loop is formed with residues 200-226 near the substrate binding site [1].

GSK-3 is associated with the pathophysiology of several diseases such as diabetes, cancer, psychiatric and neurodegenerative diseases. The GSK-3 inhibitors developed so far failed in the preclinical or clinical stages because of limited specificity and unfavorable off-target effects [2]. Tideglusib, a small molecule drug of the thiadiazolidinone (TDZD) class, is the only known GSK-3 inhibitor for which the data obtained in clinical trials support its potential use in the treatment of Progressive Supranuclear Palsy (PSP), and Alzheimer's Disease (AD) [3, 4,5]. However, recently, the Food and Drug Administration (FDA) discontinued approval of this compound because of safety and efficacy issues [6]. Some other independent studies have provided evidence that although this compound is safe, it does not provide clinical benefits, especially to mildly affected patients [7]. In most cases, clinical benefits of Tideglusib are lowered because of its inability to bind tightly and inhibit a specific drug target. In the present study, via computational methods, we performed virtual screening based on pharmacophore features using the Tideglusib structural features for identifying targetspecific GSK-3 inhibitors with better binding potentials.

## Methodology

Accelrys Discovery studio visualizer v4.0 [8] was used to visualize the receptor, ligand structures, hydrogen-bonding network, bond lengths and to render images. The ZINCPharmer online server [9] was used to identify structurally similar compounds using the pharmacophore features of Tideglusib. ArgusLab v4.0.1[10] was the primary docking program used in this study for virtual screening with semi-flexible docking. A GSK-3 receptor (PDB ID: 1PYX) and ligands were prepared for virtual screening by adding the hydrogen atoms and by removing all water molecules that were co-crystallized with this target protein. The addition of new hydrogen atoms ensures an accurate determination of the ionization states of amino acid residues. The energy of the protein structure was then minimized using the CHARMM (Chemistry at Harvard Macromolecular Mechanics) force field in the Discovery studio package, and the obtained structure was saved in a .pdb file format for further studies. Additionally, ligands were prepared by first removing the hybridization errors in the molecules and then adding the missing hydrogen bonds in order to check the fidelity of all bonds in the compounds. All prepared compounds were saved in the .mol format for further docking studies after a geometry optimization stage with the Universal Force Field (UFF) using a protocol similar to the protocols followed in previous studies [11, 12,13]. The energy-scoring grid box was centered at the active ligand binding site location and its dimensions were set to 60 Å along each axis (x, y, and z). The grid spacing was 0.40 Å, and the default atomic salvation parameters were used. The grid was assigned such that the active site of GSK-3 was enclosed by the three-dimensional grid box. “Ascore” scoring function with all docking parameters set to the default values was selected as the docking engine in ArgusLab. Following the default protocol, the details of which can be found in previous studies, [15] each molecular dynamics (MD) simulation was conducted for 20-ns using the Desmond module v3.6 [14] from Schrodinger, Inc. For a brief period, the OPLS2005 force field [16] was used to simulate the predefined three-site transferable intermolecular potential (TIP3P) water model [17]. An orthorhombic periodic boundary conditions solvent box buffered at a distance of 10 Å setup to specify the shape and size of the water simulation box. Boundary conditions box volume was calculated to be 478,000 Å3. To electrically neutralize the system, appropriate counter ions such as Cl− and Na+ were placed randomly in the solvated system.

## Results and Discussion

### Pharmacophore search

**Figure 1:**
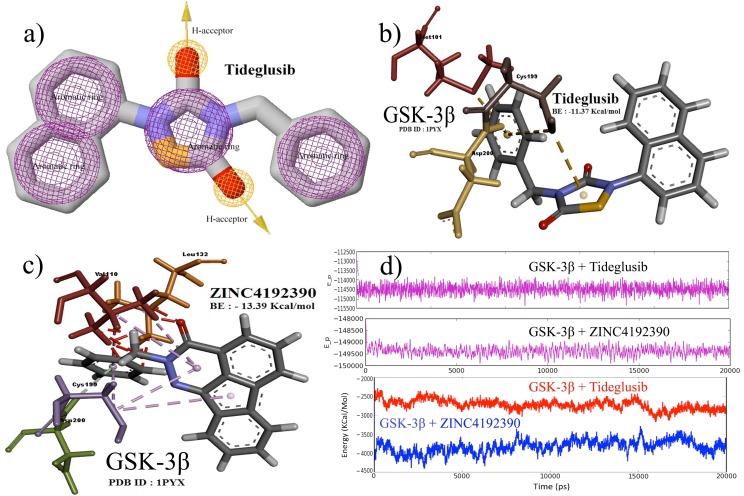
a) Pharmacophore features of Tideglusib, which was used for finding structurally similar compounds for virtual screening; b) docking snapshot of Tideglusib at the binding site of GSK-3 with a binding energy (BE) of −11.37 kcal/mol showing covalent interaction with Cys199 and hydrogen bonds with Met101 and Asp200 residues; c) docking snapshot of ZINC4192390 compound at the binding site of GSK-3 with a binding energy of −13.39 kcal/mol showing covalent interaction with Cys199 and hydrogen bonds with Val110, Leu132, and Asp200 residues; and d) MD simulation energy plots for the GSK-3 complexed with Tideglusib and ZINC4192390.

The structure of the Tideglusib compound was initially downloaded from the ZINC database (ZINC13985228) in the .mol2 format and its energy was then minimized using the CHARMM force field implemented in the Accelrys discovery studio. The obtained relaxed structure was saved in the .pdb format. The saved .pdb file was then uploaded to the ZINCPharmer online server in order to identify the pharmacophore features of the compound. Four aromatic rings with a radius of 1.10 Å were identified to be hydrophobic, and two hydrogen-bond acceptor side chains with a radius of 0.50 Å on the core aromatic ring of the compound were observed as shown in (Figure 1a). The identified pharmacophore features of the query molecule were then submitted to the online server for searching structurally similar compounds with matching pharmacophore features. A total of 416 compounds out of 17,818,291 commercially available small molecules exhibited pharmacophore features similar to those of Tideglusib; the details of these 416 compounds were saved as a database of compounds in the .sdf file format for further virtual screening studies.

### Virtual Screening

For virtual screening, a separate database comprising the above mentioned 416 compounds was created. Including Tideglusib, a total of 417 compounds were submitted for virtual screening in the ArgusLab software using the “Dock a database” option. All compounds docked in the active site of GSK-3 with binding energies ranging from -7.71 to -13.39 kcal/mol. Tideglusib was docked with a binding energy of -11.37 kcal/mol by forming a covalent interaction with Cys199 and hydrogen bonds with Met101 and Asp200, as shown in (Figure 1b). A total of 63 compounds exhibited better binding potentials (more negative binding energy) compared with that of Tideglusib. The ZINC4192390 compound was identified to be the best binding compound, with a binding energy of −13.39 kcal/mol. This high binding energy resulted from a strong covalent interaction of the Cys199 and hydrogen bonds along with pi-stacking with the Val110, Leu132, and Asp200 residues, as shown in (Figure 1c). The chemical structures of Tideglusib and ZINC4192390 are presented in the Supplementary Information.

Previous studies have reported that the Cys199 residue at the entrance of the GSK-3 active site plays a crucial role in binding Tideglusib to the receptor [18]. This hypothesis has been proved by the studies in which Tideglusib failed to show the inhibition on a panel of kinases that contain a homologous cysteine in the Cys199 location in their active sites, signifying that compounds which are able to tightly bind to this particular residue show a target specific inhibition mechanism [19]. Based on this crucial fact, it is likely that our present proposed compound ZINC4192390 (2-benzylindeno[1,2,3-de]phthalazin-3(2H)-one) has the potential to inhibit specifically target GSK-3 because the binding affinity of GSK-3 is higher than that of Tideglusib.

### MD Simulations

MD simulations were performed to confirm the binding energy and molecular level interactions determined by virtual screening. Initially, we performed individual MD simulations for GSK-3 complexed with Tideglusib and with the best compound identified via virtual screening in this study i.e., ZINC4192390 (2- benzylindeno [1,2,3-de]phthalazin-3(2H)-one). Energy plots of GSK-3 complexed with Tideglusib and with the ZINC4192390 compound for 20-ns MD simulations are shown in (Figure 1d). An inspection of the graph clearly shows that our virtual screening predictions are in good agreement with the results for the simulated receptor–ligand complexes.

According to virtual screening, the GSK-3 complexed with Tideglusib showed a binding energy of -11.37 kcal/mol whereas the GSK-3 complexed with ZINC4192390 showed a binding energy of -13.39 kcal/mol; this energy difference shows the same trend shown by the simulated energies of -114500 kcal/mol and - 149500 kcal/mol, which were observed for the total GSK-3 and Tideglusib system (protein + ligand + solvated water + ions) and for the GSK-3 and ZINC4192390 system, respectively. Therefore, it is clear that the ZINC4192390 compound exhibits a much better binding potential for GSK-3 compared with Tideglusib. Furthermore, this trend was confirmed when we analyzed the simulated energies of the GSK-3 protein in the presence of Tideglusib (red) and thereby obtained a simulated energy of - 2500 Kcal/mol. Following a similar procedure for the ZINC4192390 compound (blue), the obtained average simulated energy was -4000 Kcal/mol.

**Figure 2:**
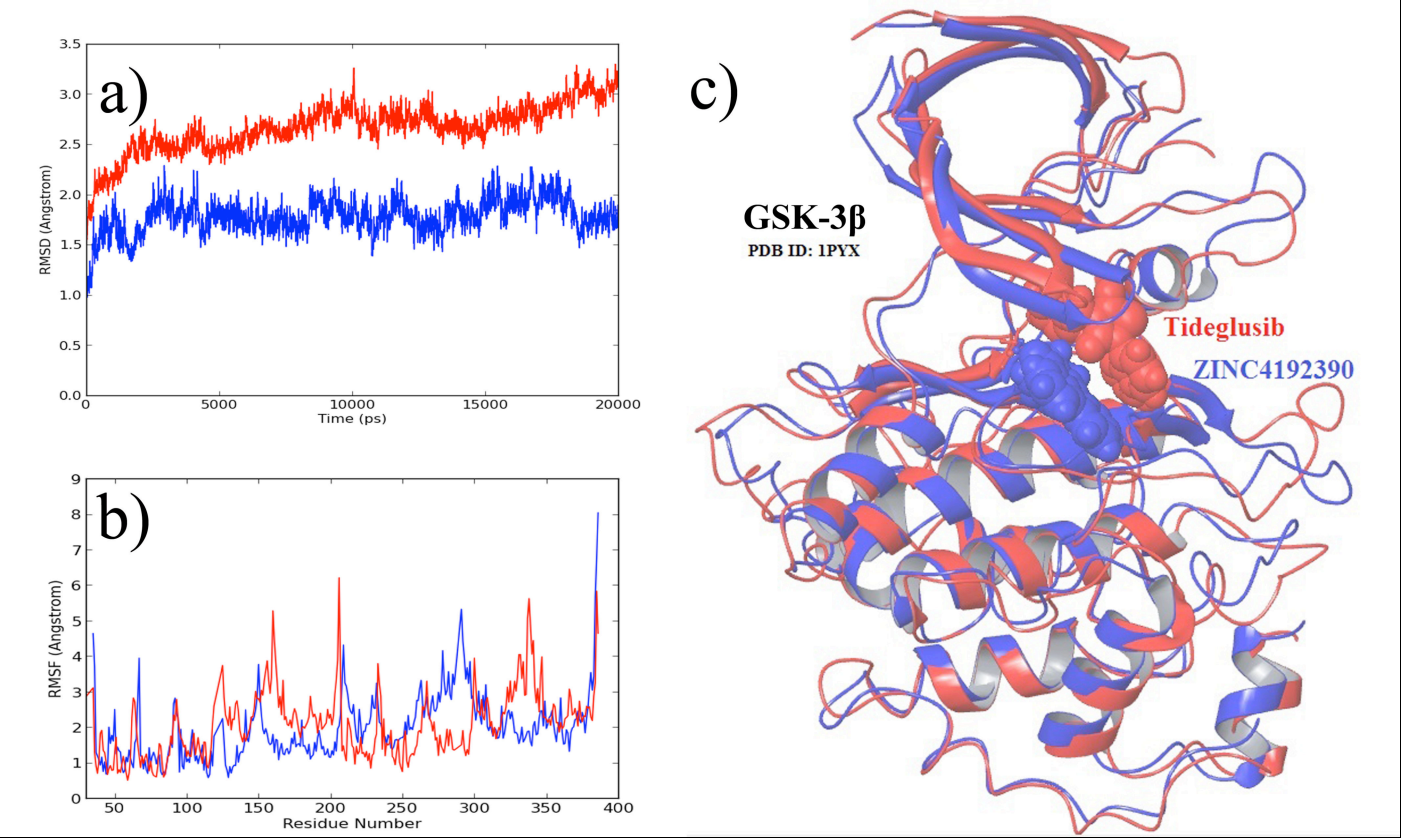
a) Root mean square deviation (RMSD) of GSK-3 protein in presence of Tideglusib (red) and in presence of ZINC4192390 compound (blue) b) Root mean square fluctuations (RMSF) of GSK-3 protein in presence of Tideglusib (red) and in presence of ZINC4192390 compound (blue) c) Superimposition of the final snapshots of MD simulations.

To further validate the stability of the GSK-3 complexed with the ZINC4192390 compound compared with that of the GSK-3 complexed with Tideglusib, we analyzed the root mean square deviation (RMSD) (Figure 2a) and the root mean square fluctuation (RMSF) (Figure 2b) of the GSK-3 protein during 20-ns MD simulations. An inspection of the graphs in (Figure 2) clearly shows that the GSK-3 complexed with ZINC4192390 is much more stable than the GSK-3 complexed with Tideglusib. Moreover, as shown in (Figure 2c), superimposition of the MD simulation snapshots revealed that the ZINC4192390 compound was located much closer to the Cys199 residue, favoring the formation of a stronger binding complex than that obtained with Tideglusib.

## Conclusion

In this study, we identified ZINC4192390 (2-benzylindeno [1,2,3- de]phthalazin-3(2H)-one) as a potential target-specific GSK-3 inhibitor. Several molecular interactions were observed for this compound, including a strong covalent interaction with the Cys199 residue at the entrance of the GSK-3 active site. This interaction plays a crucial role in the target-specific inhibition mechanism, which may have a potential to improve selectivity of the inhibition over other kinases. The results from this study are expected to provide valuable insights into strategies aimed at optimizing molecules similar to the leading Tideglusib inhibitor compound with better binding potentials and for further evaluation toward the discovery of potential GSK-3 inhibitors.

## Conflict of Interest

The authors declare that they have no conflict of interest.

## Figures and Tables

**Figure 3:**
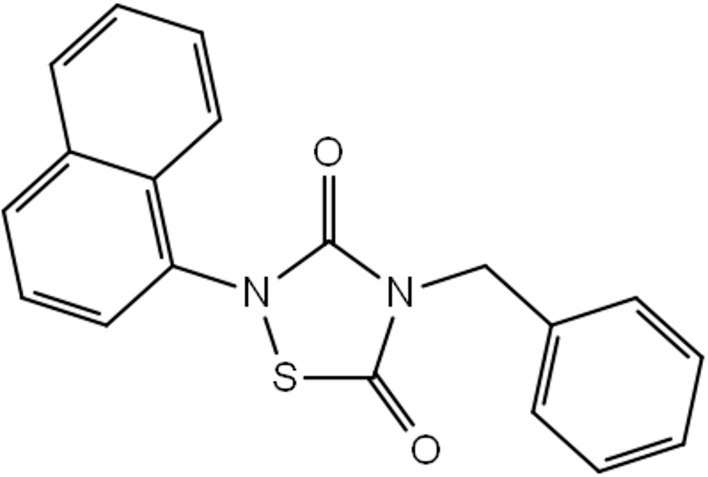
Chemical structure of Tideglusib.

**Figure 4:**
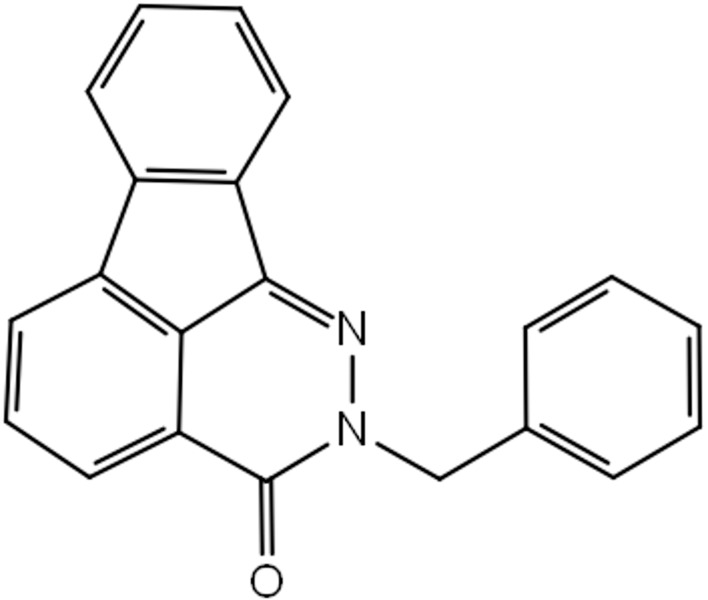
Chemical structure of ZINC4192390 (2-benzylindeno [1,2,3- de]phthalazin-3(2H)-one).
